# Zn_2_SnO_4_-Based Optoelectronic Synaptic Device for Visual Perception and Applications

**DOI:** 10.34133/research.0884

**Published:** 2025-09-09

**Authors:** Shan Xu, Zhiyuan Guan, Wenqi Yang, Zhenyu Zhou, Zixuan Zhang, Lulu Wang, Xiaoxu Li, Yuchen Li, Xiaobing Yan

**Affiliations:** College of Electron and Information Engineering, Key Laboratory of Brain-Like Neuromorphic Devices and Systems of Hebei Province, Hebei University, Baoding, People’s Republic of China.

## Abstract

Visual bionic systems are of crucial importance in the development of artificial intelligence for environmental perception. However, the traditional artificial vision system has problems such as complex system and high energy consumption due to the physical separation of image perception, storage, and processing unit. In this work, we designed an ITO/ZTO/ZnO/ITO/Mica structure optoelectronic synaptic device, which is capable of integrating optical sensing, information storage, and logic computation. Utilizing its excellent light response characteristics, the PPF, learning-experience behavior, and Pavlov experiment were successfully simulated. In a 3 × 5 array, the “F” hidden in “E” was identified by using 2 different lighting conditions, successfully simulating color recognition. Furthermore, a further design of an automatic meeting vehicle system based on the embedded platform was carried out, and the meeting vehicle behavior was successfully achieved by utilizing the light response of the ZTO device. This discovery demonstrates the potential of ZTO devices in simulating the behavior of biological synapses, providing new avenue for neuroscience research and the development of bioelectronic devices.

## Introduction

In the human perceptual system, 80% of the external information is received by the visual system [1–3]. The retina, as the front-end sensing, is responsible for receiving optical signals and preprocessing them, thus effectively removing redundant information, improving data processing efficiency, and greatly reducing system power consumption [4–6]. Inspired by this, researchers in the development of brain-like computing have proposed the use of an artificial vision system to improve the efficiency of information processing [7–9]. However, conventional machine vision systems usually consist of 3 separate units: image sensors (photodetectors), memory, and processing units [10–13]. This perception and storage separation structure not only affects the data transmission efficiency but also increases the power consumption, limiting the system performance improvement and miniaturization development. Photoelectric synapses, as emerging neuromorphic devices [14–17], show great potential in constructing artificial visual systems with perception and memory behaviors, which are expected to solve the above problems. By mimicking the plasticity of biological synapses, photoelectric synapses can realize the synergistic processing of optical information sensing, storage, and logic operations within a single device. This innovative design can effectively circumvent the data migration energy consumption problem caused by the separation of perception, storage, and computation units in the traditional artificial vision system, and at the same time significantly improve the functional integration density of the system, and provide a physical carrier for the construction of energy-efficient, miniaturized neuromorphic vision system. Compared with 3-terminal photoelectric synapses [18,19], the fabrication process of 2-terminal photoelectric synapses is simpler and the cost is lower [20–22]. Therefore, it is essential to construct optoelectronic synaptic devices that can mimic the human visual system.

Zinc–tin oxide (Zn_2_SnO_4_, ZTO), as a promising ternary oxide semiconductor material, has sparked great interest among researchers for its unique and excellent electrical and optical properties [22]. The band gap range of ZTO at room temperature is 3.3 to 3.6 eV, which features high conductivity, rapid electron transport capability, and excellent optical properties. In addition, its nontoxicity and low cost make it highly promising for a wide range of applications [23]. More importantly, the amorphous ZTO is more likely to generate more oxygen vacancies. The increase in the number of vacancies also leads to an increase in the number of trap sites for mobile ions, which is conducive to achieving synaptic plasticity and expanding the light response range to the visible light [24]. Some ionized oxygen vacancies act as shallow donors, increasing the carrier density in the ZTO channel, which in turn can enhance the peak current of the photoelectric synaptic device [22]. Therefore, ZTO materials have tremendous development potential in synaptic devices.

This work proposes an optoelectronic synaptic device based on the indium tin oxide (ITO)/ZTO/zinc oxide (ZnO)/ITO/Mica structure. This device exhibits broad-spectrum optoelectronic response characteristics in the visible light band, and its optoelectronic response characteristics can effectively simulate the behavior of visual neural synapses. The synaptic function of the device was verified by simulating PPF, learning-experience behavior, and Pavlov experiments. Furthermore, the functions of visual color recognition and visual memory have been successfully realized by using 3 × 5 arrays and 3 × 3 arrays. Based on the excellent optical response characteristics of the ZTO device, an automatic meeting system based on the embedded platform was further designed, and the meeting behavior was realized through stable optical response. These achievements not only demonstrate the great potential of ZTO materials in simulating the functions of biological synapses but also provide new ideas and directions for the future development of neuromorphic computing and intelligent perception systems.

## Results and Discussion

**Fig. 1.**
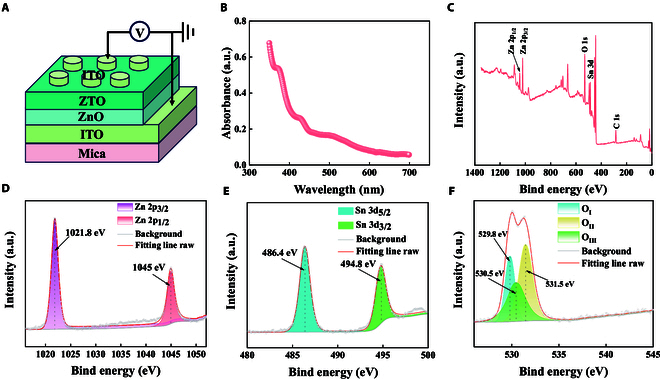
Structure and characterization of ZTO optoelectronic synaptic device. (A) Schematic diagram of the optoelectronic synaptic structure. (B) Ultraviolet–visible–near-infrared absorption spectrum. (C) XPS full spectrum of ZTO thin film. (D) Zn 2p. (E) Sn 3d. (F) O 1s.

Figure 1A illustrates the schematic structure of the optoelectronic synaptic device, which is composed of an ITO top electrode, a ZTO film, a ZnO film, an ITO bottom electrode, and a Mica substrate. The component elements are evenly distributed and have good flatness (Figs. S1 and S2). We found that the response to light varies with different thicknesses of the ZTO film (Fig. S3). The thicker the film, the greater the current response of the device. This might be because during the film growth process, as the thickness increases, the number of oxygen vacancies also increases, which in turn leads to an increase in the peak current [22]. After conducting a detailed comparative analysis of the response curves of devices of various thicknesses, it was found that the device with a thickness of 118 nm showed a relatively stable test curve, and the optical response was particularly obvious. Considering the intensity and stability of the optical response comprehensively, the device with a thickness of 118 nm was finally selected for in-depth experimental analysis. In order to study the light absorption characteristics of ZTO, the ultraviolet-visible absorption spectra were tested, mainly measuring the absorption spectra of the device in the wavelength range of 300 to 700 nm (Fig. 1B). It can be seen that the device exhibits excellent light absorption characteristics within the visible light range. The chemical properties and binding energy of the device were analyzed by x-ray photoelectron spectroscopy (XPS), and elements including Zn 2p, Sn 3d, and O 1s were detected (Fig. 1C). Figure 1D shows the Zn 2p spectrum. It can be seen that the 2 peaks correspond to Zn 2p_1/2_ and Zn 2p_3/2_, respectively, and their binding energies are 1,045 eV and 1,021.8 eV, respectively. This is caused by the energy level splitting resulting from spin–orbit coupling. The difference between the 2 peaks is 23.2 eV, which is consistent with the typical binding energy of Zn^2+^ in ZTO [25]. Based on this, the valence state of the Zn element can be determined as +2 [26]. Figure 1E shows the spectrum of Sn 3d, where the peaks at 494.8 and 486.4 eV correspond to Sn 3d_3/2_ and Sn 3d_5/2_, respectively. The difference between the 2 peaks is 8.4 eV, which is very similar to the standard spectrum of Sn 3d [27]. Figure 1F shows the spectrum of O 1s. The 3 peaks at 529.8, 530.5, and 531.5 eV belong to lattice oxygen in ZTO, vacancy oxygen, and oxygen in ZnO, respectively [28]. These laid an important theoretical foundation for the subsequent exploration of the device’s performance.

**Fig. 2.**
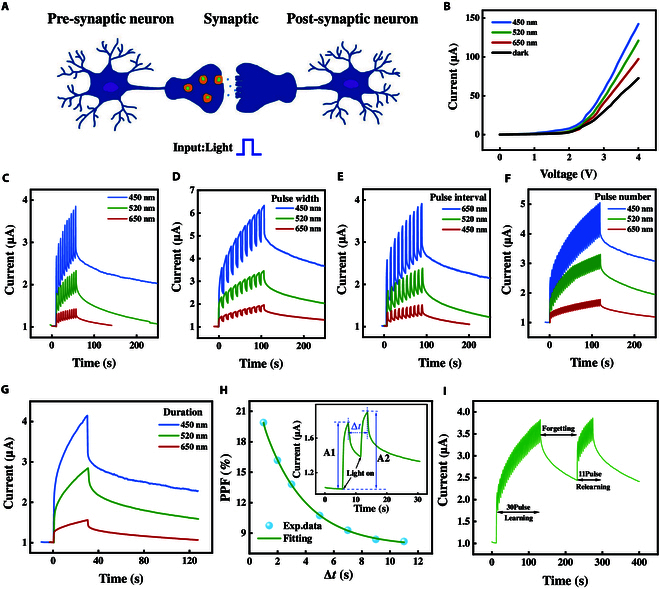
Synaptic behavior under light excitation. (A) Schematic diagram of synaptic signal conduction. (B) Current–voltage characteristic curves of the device under various lighting conditions. (C) Photocurrent responses at different wavelengths. (D) Photocurrent responses with varying pulse widths. (E) Photocurrent responses with varying pulse width intervals. (F) Photocurrent responses with varying pulse numbers. (G) Photocurrent responses with varying pulse durations. (H) Variation law of the PPF index with the pulse time interval Δ*t* and the fitting curve. (I) Current response obtained by simulating human “learning-experience” behavior through light stimulation.

In the complex neural network of the human brain, synapses play a crucial role. Their core function is to process and transmit physiological signals through the transmission of chemical signals. Similar to traditional synapses, the working principle of photoelectric synaptic device is mainly based on the conversion, transmission, and processing of optical signals. The schematic diagram of its biological structure is shown in Fig. 2A. In the optical synapse, the presynaptic neuron first responds to light stimulation and then releases neurotransmitters. These neurotransmitters bind to receptors in postsynaptic neurons, thereby enabling the transmission and processing of signals [29]. Inspired by this biological behavior, we conducted a systematic study of the device. Firstly, under various lighting conditions (darkness, 450 nm, 520 nm, 650 nm, with the power of each light being 50 mW), a forward voltage single-sided scan (0 to 4 V) was performed on the device, and its current–voltage (*I*–*V*) characteristic curve was measured (Fig. 2B).The test results show that during the voltage scanning process, the device changes from the high resistance state (HRS) to the low resistance state (LRS), demonstrating typical photoreceptor synapse characteristics, and this characteristic can be stably presented with or without light exposure. Further observation revealed that as the wavelength of light increased, the current of the device gradually decreased, indicating that it had the strongest response capability to light with a wavelength of 450 nm. This is mainly because when light shines on the ZTO/ZnO interface, new carriers are generated. These carriers are separated under the combined effect of the built-in electric field and the applied voltage, and a current increment is produced. However, the energy of different light sources varies, which leads to different total numbers of photogenerated carriers and thus results in different light response currents (Fig. S4). These characteristics have laid a solid foundation for the further in-depth study of optical synapse plasticity.

In order to simulate the function of neural synapses, the dynamic optical response characteristics of ZTO devices under different optical pulse conditions were deeply studied. By using a laser to apply light pulses of different wavelengths to the device, the current response of the device exhibits continuously changing characteristics, and this dynamic response behavior is useful for simulating the complex behavior of neural synapses. Figure 2C to G shows the photocurrent responses generated by the laser-stimulated devices with wavelengths of 650, 520, and 450 nm, respectively. When 10 optical pulses with a pulse width and an interval of 1 s are applied to the device (Fig. 2C), the current response of the device decreases as the wavelength of the laser increases. Based on Fig. 2C, further, keeping the number of optical pulses and the pulse interval unchanged, and only adjusting the pulse width to 7 s, the current response of the device is shown in Fig. 2D. It can be found that as the pulse width increases, the current value of the device also increases. While keeping other parameters unchanged and only changing the pulse interval to 7 s, the current response of the device is shown in Fig. 2E. It can be seen that as the pulse interval increases, the current value of the device shows a decreasing trend. Similarly, when the number of pulses increases to 30, the current value of the device also rises accordingly (Fig. 2F). These results indicate that the current response of the ZTO device is significantly dependent on parameters such as the wavelength, width, interval, and quantity of optical pulses, providing an important basis for its application in the simulation of neural synaptic function. Figure 2G shows the photocurrent response generated by the device after continuous exposure to light stimulation for 30 s. The data show that the photocurrents generated by the device at the 3 wavelengths are 1.56, 2.87, and 4.16 μA, respectively. Compared with the photocurrents of 1.31, 1.79, and 2.68 μA obtained from the first applied light pulse (continuous light stimulation for 1 s) in Fig. 2C, it can be found that with the extension of the duration of light stimulation, the current value of the device shows a significant increasing trend. This phenomenon indicates that the ZTO device has the ability to accumulate photogenerated carriers, thereby generating a strong conductance response and a slow conductance attenuation. This characteristic is of great significance for simulating the behavior of biological synapses and provides new possibilities for the development of neuromorphic computing and intelligent perception systems. The regulation of pulse width, interval, number of pulses, and duration for the same wavelength is shown in Figs. S5 to S7.

In biological neural networks, paired pulse facilitation (PPF) is a typical synaptic feature, manifested as an enhancement of the s excitatory postsynaptic potential (EPSP) in 2 rapidly induced closed spikes. To simulate this biological behavior, a pair of light pulses with a wavelength of 520 nm, a pulse width of 2 s, and an interval of 4s were applied to the device. The results showed that the synaptic response (A2) induced by the s stimulus was greater than that of the first stimulus (A1), which was consistent with the PPF phenomenon of biological synapses. This enhancement effect is due to the fact that after the first light pulse, the photogenerated electrons and holes are not completely collected by the electrode, resulting in the accumulation of more photogenerated carriers after the s light pulse. To quantify the change in electrical conductivity, the PPF index is used for calculation, and the formula is PPF = (A2 − A1)/A1 × 100%. Here, A1 and A2 respectively represent the current changes caused by the first and s light pulses, as shown in the inset in Fig. 2H. Then, we applied 7 sets of dual-pulse stimuli with different interval times (Δ*t*) to the ZTO device while keeping the voltage and pulse width constant. The experimental results are shown in Fig. 2H. Through formula fitting, the PPF index can be described as a function of the pulse interval Δ*t*. PPF = *K*_1_e^−Δ*t*/τ1^ + *K*_2_e^−Δ*t*/τ2^, where *K*_1_ and *K*_2_ are both the initial facilitation amplitudes, and τ_1_ and τ_2_ are the characteristic relaxation times of the fast attenuation term and the slow attenuation term, respectively. With the increase of the pulse interval Δ*t*, the PPF index gradually decreases, which indicates that the ZTO device can well simulate the PPF function of biological synapses [30]. This result verifies the potential of the ZTO device in simulating the function of biological synapses, which is of great significance for the real-time decoding and recognition of visual information [31].

The “learning-experience” behavior refers to the phenomenon that the human brain takes less time to relearn and recall the learned content than the initial learning time [31]. This is closely related to the plasticity of synapses in the brain and plays a key role in the learning and adaptation process of the brain. In this study, we stimulated the ZTO device with light pulses of a wavelength of 520 nm to simulate this “learning-experience” behavior. Figure 2I shows that during the initial learning stage of 30 light pulses, the photocurrent response of the device significantly increases and then spontaneously decays to a transitional state during the learning intervals. This may be similar to the learning and forgetting processes in the human brain [32,33]. In the s stimulus (relearning) stage, only 11 light pulses are needed to restore the response current of the device to the state after the initial learning, which is significantly reduced compared to the 30 pulses required for the initial learning. This phenomenon is similar to human learning and memory behavior, that is, the time required for relearning is shorter than that for the initial learning. It can be known from this that ZTO devices can exhibit “learning-experience” behavior in the form of photoelectric synaptic device. To prove that the ZTO device has the ability of interactive associative learning, we simulated Pavlov’s classical conditioned reflex experiment. Five light pulses with a wavelength of 520 nm, a pulse width of 1 s, and an interval of 2 s were applied to simulate the stimulation brought by ringing, and 5 light pulses with a wavelength of 450 nm, a pulse width of 1 s, and an interval of 2 s were applied to simulate the stimulation brought by food. Simultaneously applying light pulses of 520 and 450 nm to simulate the superposition of the shock bell and food stimulation (Fig. S8), the ZTO device demonstrated a good light response and successfully simulated Pavlov’s conditioned reflex experiment, which was consistent with Pavlov’s theory. The results show that the ZTO device successfully simulates the 2-photon integration characteristics of biological synapses through the synergistic stimulation of 520- and 450-nm optical pulses. The energy coupling of heterogeneous photons triggers the dynamic adjustment of synaptic weights to achieve synaptic connection enhancement, accurately reproducing the “co-activation equals enhancement” mechanism in the Hebbian learning rule, providing a physical implementation basis for the development of electro-optical fusion brain-like devices.

**Fig. 3.**
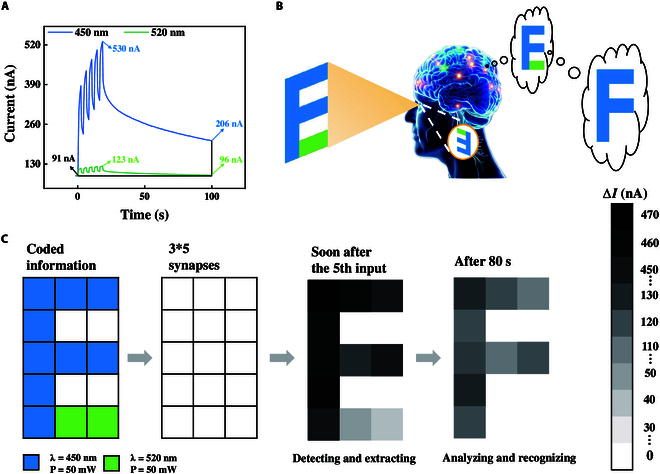
Simulated color recognition. (A) Current responses of the device under 5 optical pulses with wavelengths of 450 and 520 nm. (B) Schematic diagram of the human visual system’s recognition of color patterns. (C) Color pattern recognition similar to the human visual system achieved through memristors.

In addition, we also utilized devices with an 80-nm thickness to study the color recognition characteristics. We achieved simple color differentiation through the physical structure and applied it to the computation of information encryption. The experiment found that the ZTO device with a thickness of 80 nm exhibited different photoconductance behaviors under optical pulses with wavelengths of 450 and 520 nm (Fig. S9). It showed nonvolatile photoconductance under 450-nm photoexcitation, while it exhibited volatile photoconductance under 520-nm optical pulses. This characteristic enables the device to effectively distinguish light stimuli of different wavelengths and simulate the human eye’s color recognition behavior. Figure 3A shows the response comparison of the device to optical pulses with wavelengths of 450 and 520 nm. When the initial current is 91 nA, 5 optical pulses with wavelengths of 450 and 520 nm, respectively, and each pulse width and interval is 2 s, are applied. After 5 optical pulses, the response currents of the device to 450- and 520-nm light increased to 530 and 123 nA, respectively, and after 100 s, the response currents decreased to 206 and 96 nA, respectively. It can be seen that the device has a higher current response to 450-nm optical pulses and still maintains a relatively high level after 100 s. However, the response to the 520-nm light pulse is relatively small and basically returns to the initial value after 100 s. Since the absorption intensity of 450-nm light is greater than that of 520-nm light, under the same power, the response current of this device to blue light is greater than that to green light, which means that more carriers are generated. Moreover, due to the defects in the device itself, the total number of carriers produced by 520-nm light is less. Under the same conditions, when carriers undergo recombination, they are consumed more quickly. Therefore, there are fewer remaining carriers and the accumulated number of carriers is limited. The light response shows volatile characteristics. However, 450-nm light generates more carriers, and under the same recombination ability, the remaining carriers are relatively more. The excess carriers will accumulate at the electrode interface, thereby causing nonvolatile characteristics. This differentiated response can simulate the behavior of the human eye in recognizing colors. Figure 3B is a schematic diagram of the process by which the human brain acquires information from the outside world through the visual system, including light information detection through the eyes, color extraction by the retina, and information analysis by the central visual system. The retina does not simply convey information based on the light and dark patterns that fall on it [31]. Instead, it extracts information of different colors and transmits it to the central visual system in the occipital lobe, enabling the blue part “F” in the letter “E” to be accurately recognized by the human brain.

Based on the unique optical response of the ZTO device, biological behaviors can be simulated. Fifteen photoelectric synaptic devices arranged in a 3 × 5 configuration is selected from the ZTO device to identify and store images. By applying light pulses with wavelengths of 450 or 520 nm and a pulse width and interval of 2 s to different devices in the array, a letter “F” composed of the blue part and a horizontal line “_” composed of the green part were formed. These 2 parts then formed the letter “E”, as shown in Fig. 3C. In this test, the postsynaptic current response was obtained with a reading voltage of 500 mV. First, the synaptic array detects the letter “E” with different brightness levels and cannot clearly distinguish between blue and green. This is very similar to the process by which the human eye transmits the detected information to the retina [31]. However, after 100 s, the synaptic array still had a relatively high current response to the blue “F” part in the detected letter “E”, while the current response to the green “_” part was almost zero. It could clearly distinguish between the blue and green parts, similar to the operation mode of the retina and the central visual system, and could effectively extract and analyze color information. Furthermore, a photoelectric synaptic plasticity based on the same light stimulation but with different readout voltages was also designed (Fig. S10). All of these demonstrate that this device has certain application potential in the field of neuromorphic computing.

**Fig. 4.**
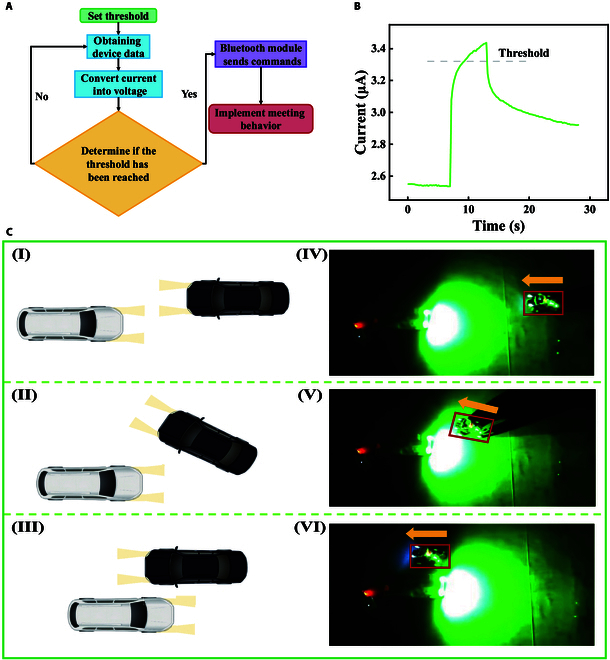
Meeting oncoming vehicles at night system. (A) Flowchart of the meeting system. (B) Test diagram of the photocurrent response of the ZTO device during the meeting process, with the dotted line representing the set threshold. (C) Schematic diagram of the meeting and the actual test screen.

Photoelectric synaptic devices also show promising applications in the field of smart driving. Meeting oncoming vehicles at night is a common behavior in the process of automobile driving, and the improper use of high beams in the process of meeting vehicles may lead to traffic accidents. Through effective proactive avoidance actions, it can reduce the accidents caused by improper use of high beams. Based on the light synaptic response characteristics of ZTO devices, a meeting vehicles electronic system has been designed here. This system mainly utilizes photoelectric synaptic device, which can continuously increase the photocurrent under continuous illumination. Once the current reaches the system’s threshold, the vehicle will perform a steering avoidance action. Therefore, we have designed an embedded platform, and its circuit structure is shown in Fig. S11. It mainly includes ZTO devices, signal generators, conversion modules, microcontroller systems, and Bluetooth modules. Among them, the signal generator supplies voltage to the ZTO device. The conversion module converts the measured current value into a voltage value. The microcontroller system collects the voltage value and determines whether it reaches the threshold. Finally, it sends the corresponding meeting instructions to the car through the Bluetooth module. The meeting vehicle process is shown in Fig. 4A. Firstly, a threshold is set using the microcontroller system (Fig. 4B). Then, the response values are continuously monitored. By utilizing the continuous response characteristics of the photo-sensitive transistor to light, when light from the approaching vehicle continuously shines (Fig. 4C, I and IV), due to the continuous accumulation of current, once the system detects a situation exceeding the set threshold, a steering command will be issued to control the vehicle to perform the overtaking action (Fig. 4C, II and V). Once the overtaking action is completed, the vehicle will return to its normal driving state (Fig. 4C, III and VI). Our simulation verification experiments have preliminarily confirmed that the photonic synapse device can effectively enhance the safety of intelligent assisted driving applications.

## Conclusion

In this work, we propose an optical synapse device based on ITO/ZTO/ZnO/ITO/Mica, providing a new idea for the hardware implementation of artificial vision systems. Through the timing regulation of optical pulses, this device successfully simulated behaviors such as PPF, learning-experience behavior, and Pavlov experiments. Furthermore, the visual color recognition function has also been implemented in the 3 × 5 device array. Based on the optical response stability of the device, an automatic meeting oncoming vehicles at night system based on the embedded platform was further constructed. The meeting behavior in the autonomous driving scenario was successfully simulated through real-time interaction of optical signals. This design strategy that integrates the synaptic plasticity mechanism with visual sensing provides an extensible hardware platform for the development of artificial vision systems with environmental adaptive capabilities.

## Materials and Methods

The main materials used in the ZTO photoreceptor synapse device in this article are Mica substrate, ZnO, ZTO (Zn_2_SnO_4_), and ITO (In_2_O_3_:SnO_2_ = 9:1). On the Mica substrate, ITO substrate electrodes, ZnO, and ZTO films were deposited successively by magnetron sputtering. The operation steps are as follows. The specific steps for preparing the ITO electrode are as follows: After the pressure reaches 1.8 × 10^−4^ Pa, the flow rate of argon and oxygen was set at 5:1, the gate valve was adjusted, the pressure was increased to 0.5 Pa, and the sputtering power was set to 100 W. After the presputtering, a formal sputtering for 60 min can be carried out to complete the preparation of the ITO electrode. It should be noted that the conditions for the bottom electrode and the top electrode are the same, but the top electrode needs to be grown in conjunction with a mask plate with a diameter of 100 μm. Then, ZTO films are grown by radio frequency magnetron sputtering. The vacuum at the back of the equipment is 2 × 10^−4^ Pa, the ratio of Ar to oxygen is 1:2, the sputtering power is 80 W, the sputtering pressure is 3 Pa, and the sputtering duration is 30 min. The back pressure of the magnetron sputtering equipment is pumped to 2 × 10^−4^ Pa, and then, the argon oxygen ratio inside the cavity is modulated to 1:2. The preparation of ZnO thin films is completed under the conditions of a sputtering power of 80 W and a sputtering pressure of 3 Pa. The sputtering duration is 10 min.

## Data Availability

The data that support the findings of this study are available from the corresponding authors upon reasonable request.
