# Supplementary material for: Zn2SnO4-Based Optoelectronic Synaptic Device for Visual Perception and Applications
Source: Research (Wash D C). 2025 Sep 9;8:0884. doi: 10.34133/research.0884 (PMC12417634; doi:10.34133/research.0884)
Supplement: Supplementary 1 — Figs. S1 to S11 [file research.0884.f1.docx]

**Supplementary Material**

**Zn_2_SnO_4_-Based Optoelectronic Synaptic Device for Visual Perception and Applications**

Shan Xu^1†^, Zhiyuan Guan^1†^,Wenqi Yang^1^, Zhenyu Zhou^1^*, Wang Jialin^1^, Zixuan Zhang^1^, LuluWang^1^, Xiaoxu Li^1^, Yuchen Li^1^, Xiaobing Yan^1^*

1 College of Electron and Information Engineering, School of Life Sciences, Institute of Life Science and Green Development, Key Laboratory of Brain-Like Neuromorphic Devices and Systems of Hebei Province, Hebei University, Baoding, the People's Republic of China.

* Corresponding author. E-mail: [xiaobing_yan@126.com](mailto:xiaobing_yan@126.com) and zhenyu_zhou@126.com

†Shan Xu and Zhiyuan Guan contributed equally to this work


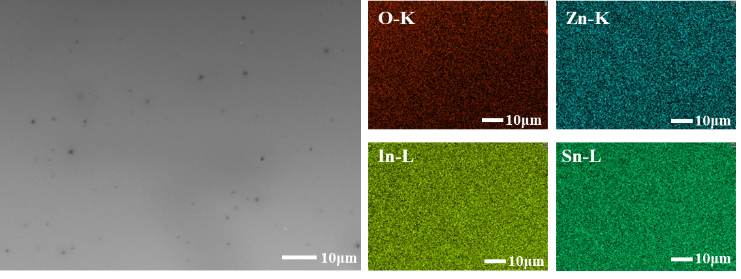


Figure S1 SEM and EDS mapping images of the sample surface.


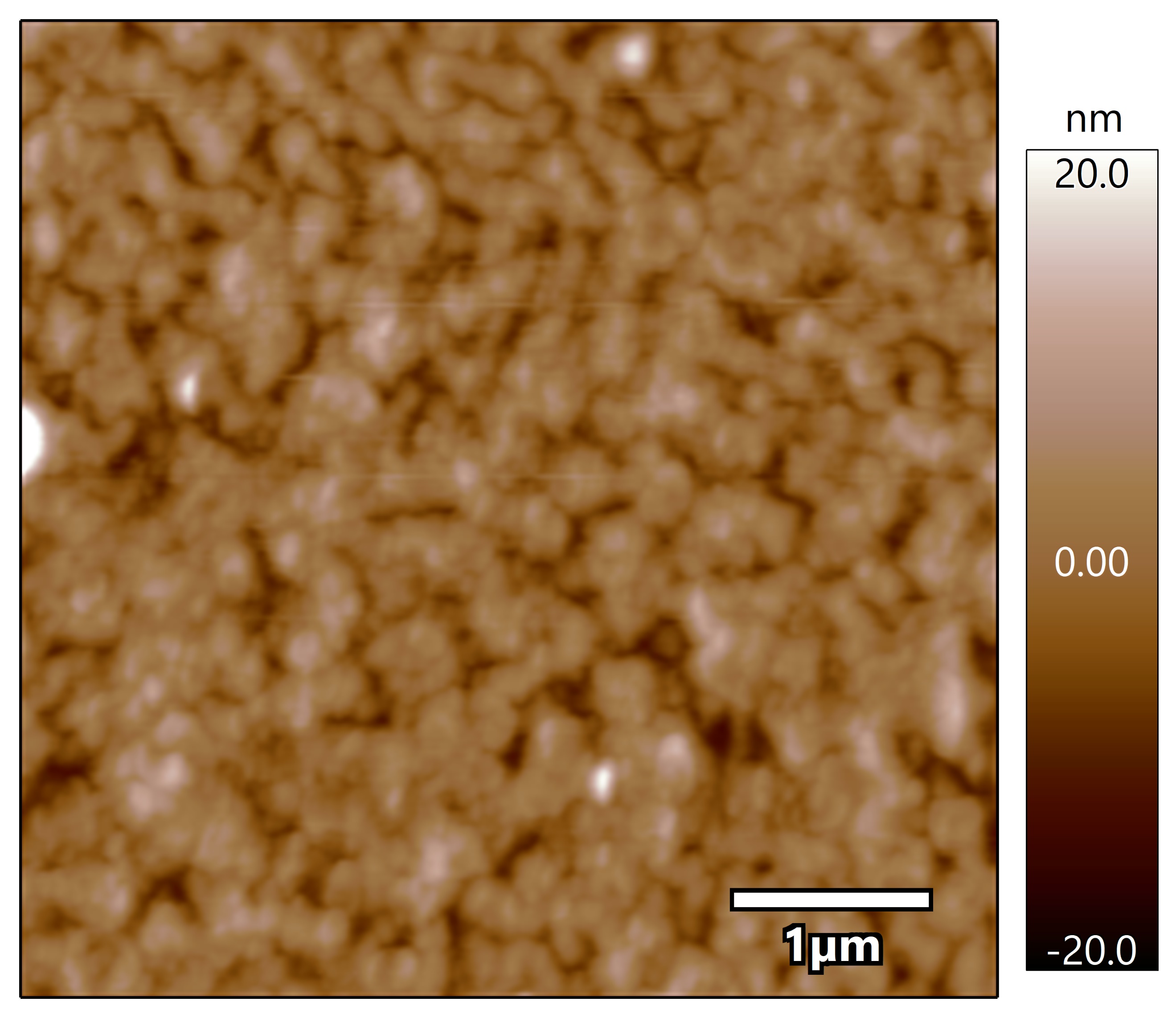


Figure S2 The AFM on the surface of the device


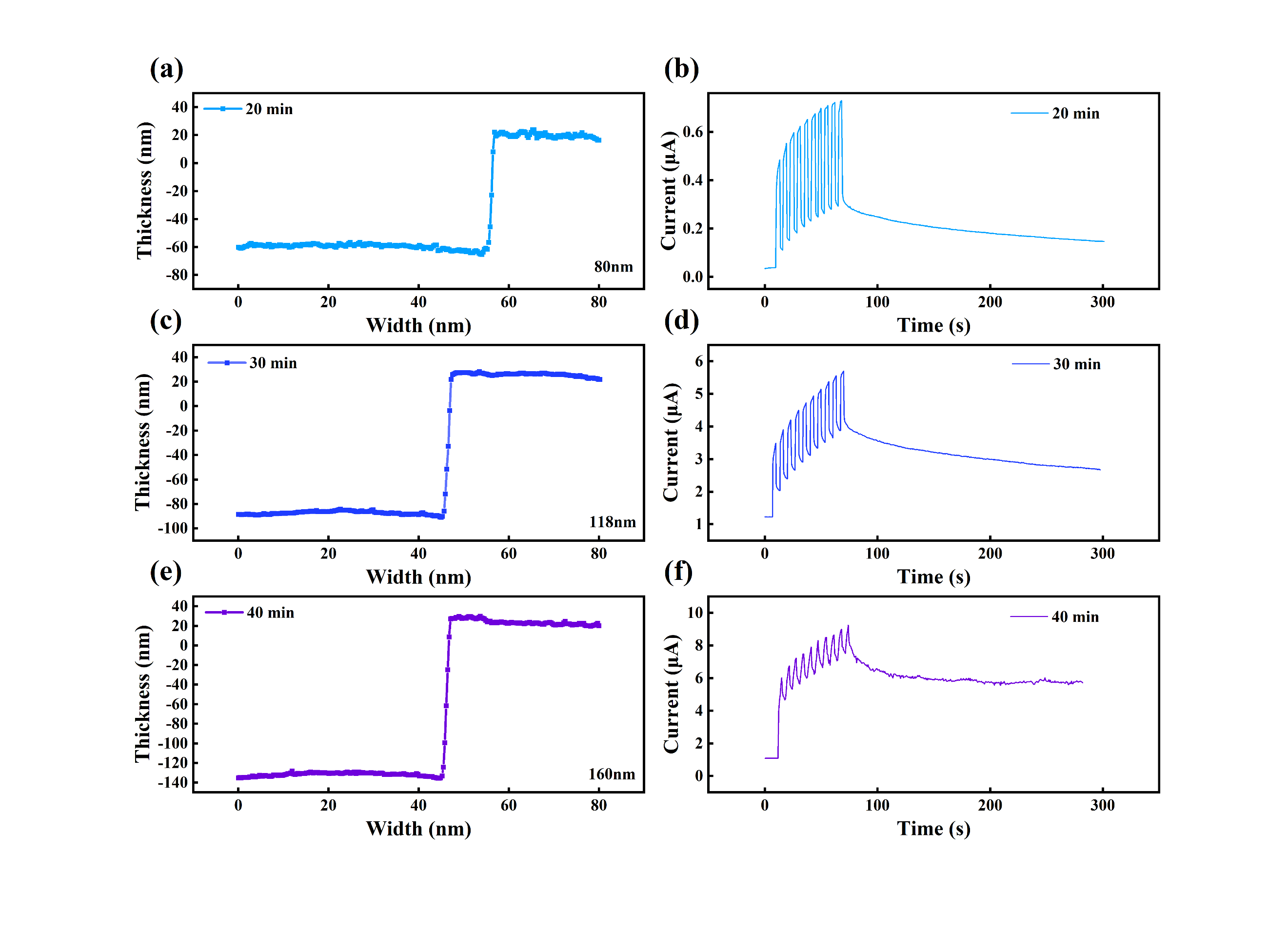


**Figure S3.** Effect of ZTO film thickness on the optical response of the device. (a) film thickness case for sputtering time of 20 min (80 nm). (b) light pulse response case for sputtering time of 20 min. (c) film thickness case for sputtering time of 30 min (118 nm). (d) light pulse response case for sputtering time of 30 min. (e) film thickness case for sputtering time of 40 min (160 nm). (f) light pulse response case for sputtering time of 40 min for the light pulse response.


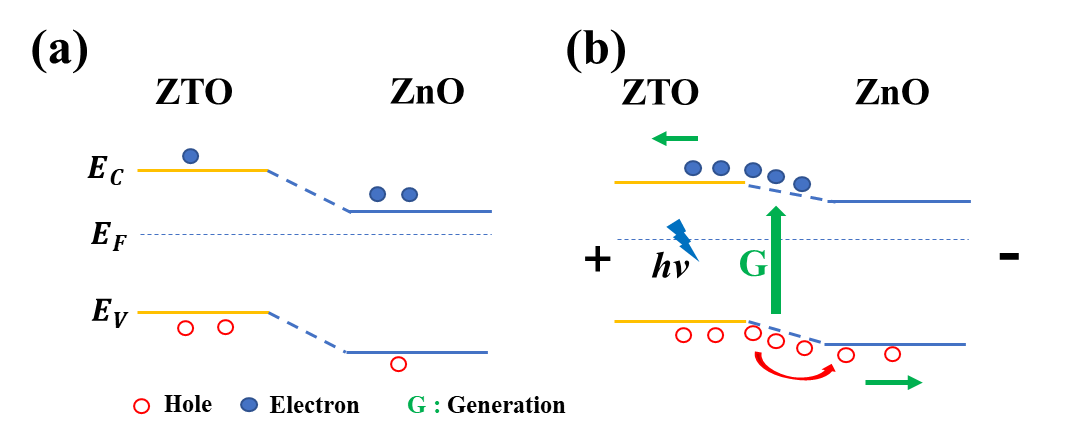


**Figure. S4** (a) Band diagram in the state of thermal equilibrium; (b) Band diagram under photoelectric action.

Under a thermal equilibrium state, since the electron affinity of ZnO is higher than that of ZTO, the electrons in ZTO will transfer to ZnO, resulting in a positive charge at the ZTO interface and a negative charge at the ZnO interface. This leads to the formation of an internal electric field and potential barrier at the interface (Figure S4a). When visible light shines on the ZTO/ZnO interface, electron-hole pairs are generated at the interface. Under the combined effect of the built-in electric field and the applied voltage, electrons migrate to the ZTO side and holes migrate to the ZnO side. The charge distribution at the interface changes, and the height of the potential barrier also changes (Figure S4b). Under light excitation, the generated holes are captured by the traps at the ZTO/ZnO interface, causing charge accumulation, a decrease in the potential barrier height, and an increase in the conductivity of the device. After the light stops, the captured holes will gradually recombine, and this process can generate the memory characteristic of the light signal within a relatively short period of time.


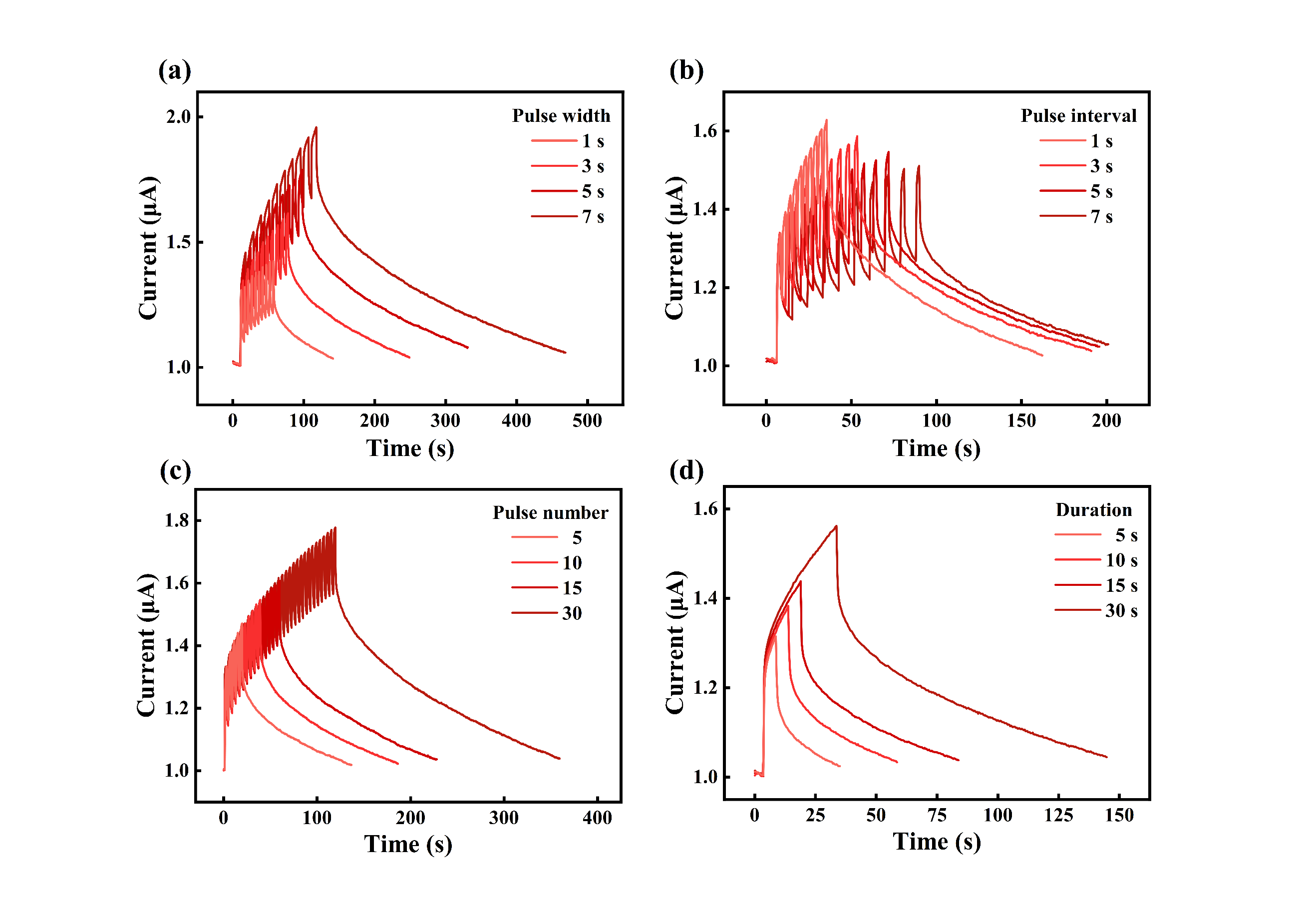


**Figure S5.** Photocurrent responses of ITO/ZTO/ZnO/ITO/Mica devices under 650 nm wavelength light. (a) Photocurrent responses curves obtained by varying the pulse width of the light pulses. (b) Photocurrent responses curves obtained by varying the spacing of the light pulses. (c) Photocurrent responses curves obtained by varying the number of light pulses. (d) Photocurrent responses curves obtained by varying the irradiation time Characteristic curves.


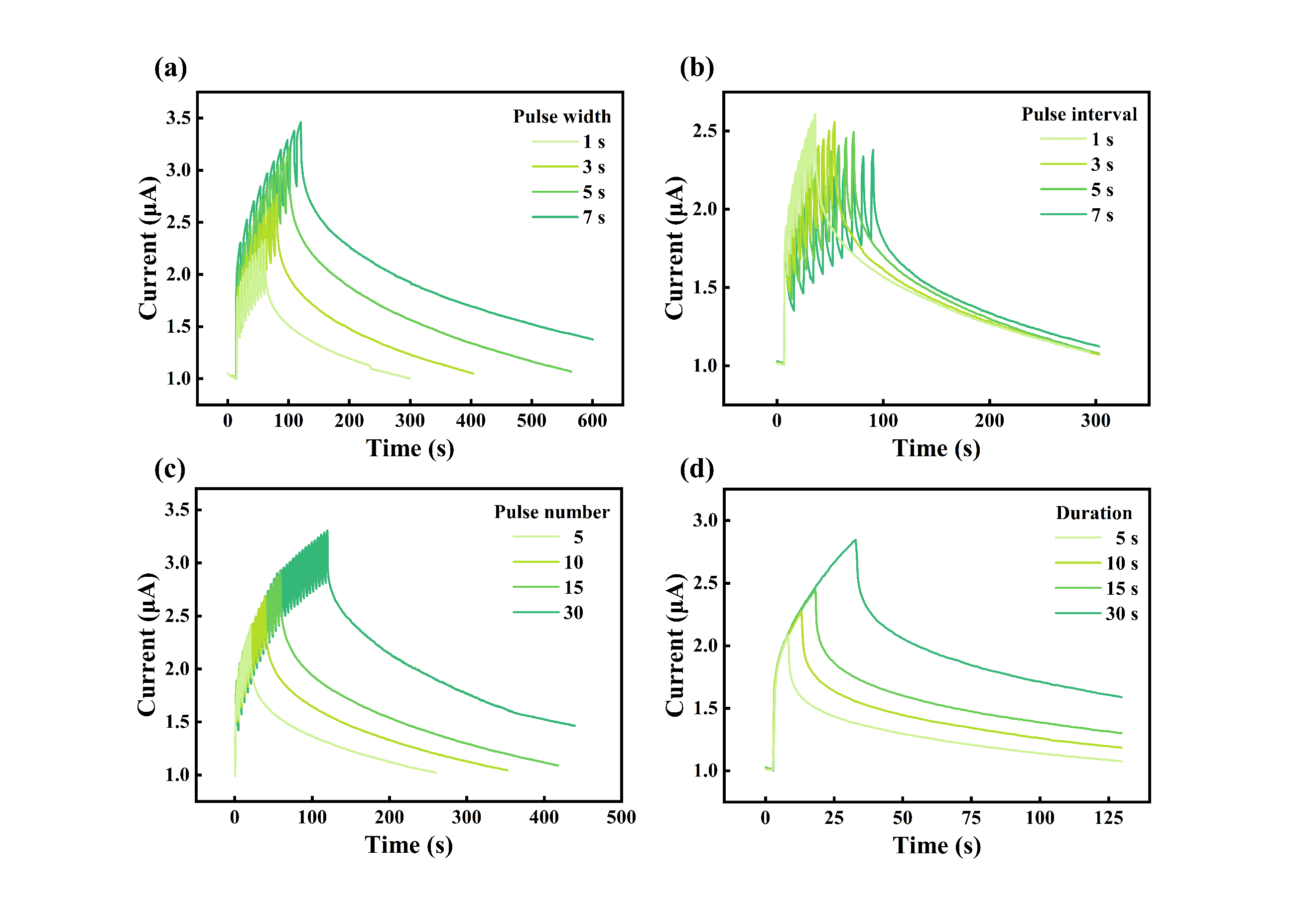


**Figure S6.** Photocurrent responses of ITO/ZTO/ZnO/ITO/Mica devices under 520 nm wavelength light. (a) Photocurrent responses curves obtained by varying the pulse width of the light pulses. (b) Photocurrent responses curves obtained by varying the spacing of the light pulses. (c) Photocurrent responses curves obtained by varying the number of light pulses. (d) Photocurrent responses curves obtained by varying the irradiation time.


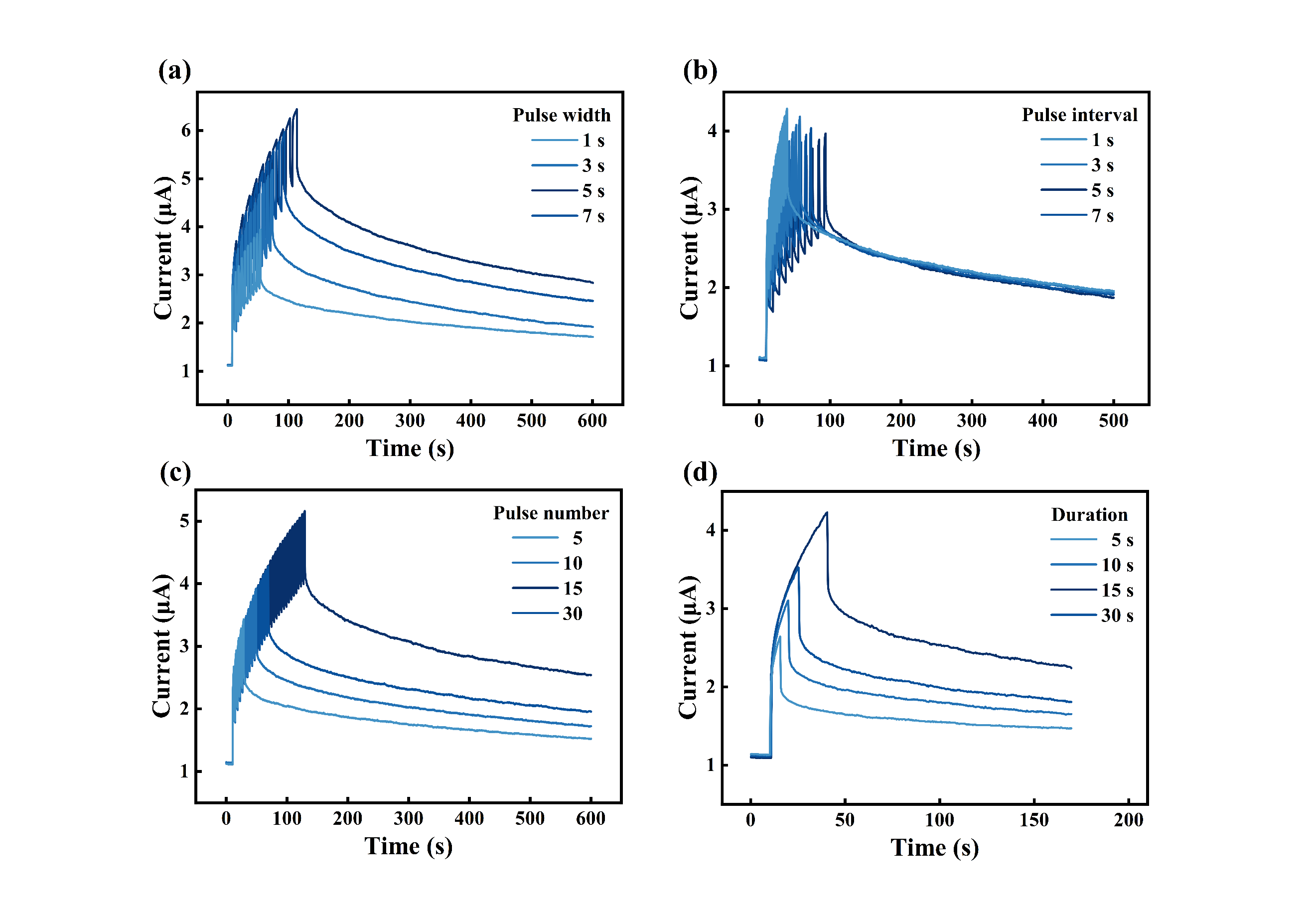


**Figure S7.** Photocurrent responses of ITO/ZTO/ZnO/ITO/Mica devices under 450 nm wavelength light. (a) Photocurrent responses curves obtained by varying the pulse width of the light pulses. (b) Photocurrent responses curves obtained by varying the spacing of the light pulses. (c) Photocurrent responses curves obtained by varying the number of light pulses. (d) Photocurrent responses curves obtained by varying the irradiation time.

**Figure S8.** (a) Using 520 nm light as a conditioned stimulus. (b) Using 450 nm light as an unconditioned stimulus. (c) Simulating the training phase using both 520 nm and 450 nm light stimuli. (d) Using 520 nm light again as a post-training stimulus. with the gray dashed line indicating that the threshold was set to 2.29 µA

**Figure S9.** Photocurrent responses of ZTO devices with a film thickness of 80 nm at 450 nm (a-c) and 520 nm (d-e) wavelengths. (a. d) Photocurrent responses curves obtained by varying the pulse widths of the light pulses. (b. e) Photocurrent responses curves obtained by varying the intervals of the light pulses. (c. f) Photocurrent responses curves obtained by varying the number of light pulses.

**Figure S10.** (a) Current response of the device to 10 light pulses at different reading voltages. (b) Current response of the device to 20 s of sustained light at different reading voltages. (c) Histogram of the current response amplitude ΔI to 20 s of sustained light at different reading voltages. (d) Statistics to mimic adjustable human visual memory by applying different reading voltages.

Human visual memory is the ability of the brain to process, store and recall visual information. Visual memory can be divided into short-term memory (STM) and long-term memory (LTM), corresponding to different storage locations and maintenance durations respectively. We designed a 3×3 synaptic array for image recognition and storage. Under the same conditions, increasing the reading voltage under the stimulation of optical pulses can lead to a persistent photoconductive behavior of the optical synapse with a long attenuation time (Figure S10 (a)). The photocurrent response tests of the device under different reading voltages for 20 s under light exposure at a wavelength of 450 nm are shown in Figure S10(b). It can be clearly observed from the figure that with the increase of the applied voltage, the photocurrent response of the device increases linearly. Figure S10(c) shows the comparison data of the current response amplitude ΔI. The tunable characteristics of this photocurrent response can be attributed to the increase in carrier transport speed under bias voltage and the decrease in the recombination efficiency of electron-hole pairs. This characteristic enables the optical synapse to simulate the iris for light adaptation, ensuring stable working performance under different lighting conditions. Figure S10(d) shows the "cross" images recorded by the 3×3 array at reading voltages of 100 mV and 500 mV respectively. The voltages of 100 mV and 500 mV correspond respectively to the low-interest and high-interest responses of the same input image. Just as expected, under the same illumination, the "cross" in the image obtained with a reading voltage of 500 mV is more clearly visible than that in the image obtained with a reading voltage of 100 mV. This proves that the photoelectric synaptic device based on ZTO have great potential in the application of neuromorphic computing and advanced robot vision systems.


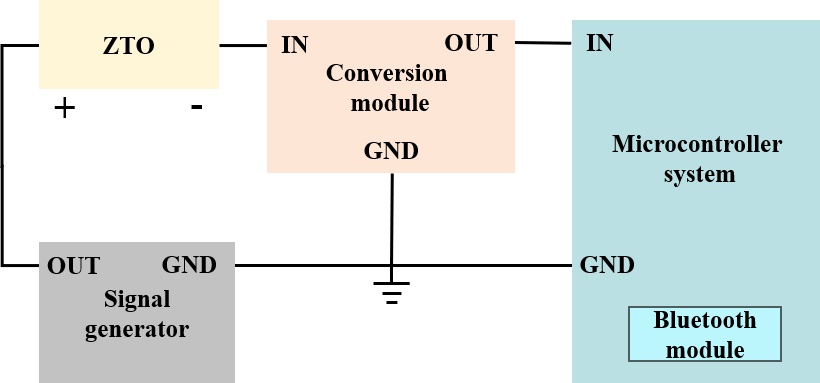


**Figure S11.** Rendezvous System Circuit Diagram.
